# The Barrett‐associated variants at *GDF7* and *TBX5* also increase esophageal adenocarcinoma risk

**DOI:** 10.1002/cam4.641

**Published:** 2016-01-18

**Authors:** Jessica Becker, Andrea May, Christian Gerges, Mario Anders, Claudia Schmidt, Lothar Veits, Tania Noder, Rupert Mayershofer, Nicole Kreuser, Hendrik Manner, Marino Venerito, Jan‐Hinnerk Hofer, Orestis Lyros, Constantin J. Ahlbrand, Michael Arras, Sebastian Hofer, Sophie K. M. Heinrichs, Katharina Weise, Timo Hess, Anne C. Böhmer, Nils Kosiol, Ralf Kiesslich, Jakob R. Izbicki, Arnulf H. Hölscher, Elfriede Bollschweiler, Peter Malfertheiner, Hauke Lang, Markus Moehler, Dietmar Lorenz, Katja Ott, Thomas Schmidt, Markus M. Nöthen, Andreas Hackelsberger, Brigitte Schumacher, Oliver Pech, Yogesh Vashist, Michael Vieth, Josef Weismüller, Michael Knapp, Horst Neuhaus, Thomas Rösch, Christian Ell, Ines Gockel, Johannes Schumacher

**Affiliations:** ^1^ Institute of Human GeneticsUniversity of BonnBonnGermany; ^2^ Department of GenomicsLife & Brain CenterUniversity of BonnBonnGermany; ^3^ Department of Medicine IISana KlinikumOffenbachGermany; ^4^ Department of Internal Medicine IIEvangelisches KrankenhausDüsseldorfGermany; ^5^ Department of Interdisciplinary EndoscopyUniversity Hospital Hamburg‐EppendorfHamburgGermany; ^6^ Departments of Gastroenterology and Interdisciplinary EndoscopyVivantes Wenckebach‐KinikumBerlinGermany; ^7^ Department of General, Visceral and Cancer SurgeryUniversity of CologneCologneGermany; ^8^ Institute of PathologyKlinikum BayreuthBayreuthGermany; ^9^ Gastroenterologie am BurgweiherBonnGermany; ^10^ Department of Visceral TransplantThoracic and Vascular SurgeryUniversity Hospital of LeipzigLeipzigGermany; ^11^ Department of Internal Medicine IIHSK HospitalWiesbadenGermany; ^12^ Department of GastroenterologyHepatology and Infectious DiseasesOtto‐von‐Guericke University HospitalMagdeburgGermany; ^13^ Magen Darm Zentrum Wiener PlatzCologneGermany; ^14^ Department of GeneralVisceral and Transplant SurgeryUniversity Medical CenterUniversity of MainzMainzGermany; ^15^ Department of General, Visceral and Thoracic SurgeryUniversity Medical Center Hamburg‐EppendorfUniversity of HamburgHamburgGermany; ^16^ First Department of Internal MedicineUniversity Medical CenterUniversity of MainzMainzGermany; ^17^ Departments of General and Visceral SurgerySana KlinikumOffenbachGermany; ^18^ Department of General, Visceral and Transplantation SurgeryUniversity of HeidelbergHeidelbergGermany; ^19^ Department of General, Visceral and Thorax SurgeryRoMed Klinikum RosenheimRosenheimGermany; ^20^ GastropraxisWiesbadenGermany; ^21^ Departments of Internal Medicine and GastroenterologyElisabeth HospitalEssenGermany; ^22^ Departments of Gastroenterology and Interventional EndoscopySt. John of God HospitalRegensburgGermany; ^23^ Gastroenterologische GemeinschaftspraxisKoblenzGermany; ^24^ Institute for Medical BiometryInformatics and EpidemiologyUniversity of BonnBonnGermany

**Keywords:** Esophageal adenocarcinoma, genetic association study, *TBX5*, *GDF7*, *ALDH1A2*

## Abstract

Barrett's esophagus (BE) and esophageal adenocarcinoma (EAC) represent two stages within the esophagitis‐metaplasia‐dysplasia‐adenocarcinoma sequence. Previously genetic risk factors have been identified that confer risk to BE and EAC development. However, to which extent the genetic variants confer risk to different stages of the BE/EAC sequence remains mainly unknown. In this study we analyzed three most recently identified BE variants at the genes *GDF7* (rs3072), *TBX5* (rs2701108), and *ALDH1A2* (rs3784262) separately in BE and EAC samples in order to determine their risk effects during BE/EAC sequence. Our data show that rs3072 at *GDF7* and rs2701108 at *TBX5* are also associated with EAC and conclude that both loci confer disease risk also at later stages of the BE/EAC sequence. In contrast, rs3784262 at *ALDH1A2* was highly significantly associated with BE, but showed no association with EAC. Our data do not provide evidence that the *ALDH1A2* locus confers equal risk in early and late stages of BE/EAC sequence.

## Introduction

Barrett's esophagus (BE) is characterized by replacement of squamous epithelium by metaplasic columnar epithelium and represents a common premalignant condition affecting 1–2% of the adult population in Western developed countries 1. Individuals with BE have a 2–4% life time risk of esophageal adenocarcinoma (EAC) 2, which presents the end point in the esophagitis‐metaplasia‐dysplasia‐adenocarcinoma sequence. Chronic inflammation due to gastroesophageal reflux disease (GERD) is the predominant etiologic factor for BE 3. GERD and thereby BE risk is further influenced by hiatal hernia and obesity 3. In addition, genetic factors play a role in BE and EAC development. So far, two genome‐wide association studies (GWAS) for BE have been published. Su et al. used a discovery sample of 1852 BE cases and 5172 controls as well as several replication samples (total of 5986 patients and 12,825 controls) 4. They identified genome‐wide significant BE association at the HLA‐region on chromosome 6p21 and near *FOXF1* on chromosome 16q24. In a following study using 318 patients and 431 controls it has been shown that both loci confer also EAC risk 5. The second GWAS by Levine et al. used a combined discovery BE/EAC sample consisting of 2416 BE cases and 1516 EAC cases as well as 3209 controls 6. Their best findings were followed up in a replication sample (1633 BE/EAC patients and 6911 controls), which lead to genome‐wide significant associations near *CRTC1* on chromosome 19p13, *BARX1* on chromosome 9q22 and *FOXP1* on chromosome 3p13. Except for the finding at *CRTC1* the associations near *BARX1* and *FOXP1* have been independently replicated in a sample of 1065 EAC cases and 1019 controls 7.

In addition to these loci, three BE associations have been published most recently 8. The replication of the GWAS in BE by Sue et al. was extended to a total of 8306 BE cases and 15,890 controls, in which additional 65 prioritized SNPs from the discovery phase were genotyped 4. This lead to genome‐wide significant BE associations at rs3072 on chromosome 2p24 and at rs2701108 on chromosome 12q24 8. Within the chromosomal 2 region *GDF7* represents the most promising risk gene and *TBX5* within the chromosomal 12 region. In addition, the authors performed a meta‐analysis using both published GWAS datasets and followed up the most significant associations in their replication samples. This lead to an additional genome‐wide significant BE association at rs3784262 on chromosome 15q22 with *ALDH1A2* being the most promising risk gene at this locus 8.

In this study, we aimed at replicating the observed BE associations at *GDF7*,* TBX5,* and *ALDH1A2*. In addition, we tested whether the implicated loci are also conferring risk to EAC and if so, whether the risk effects differ between BE and EAC.

## Material and Methods

Our sample consisted of 542 BE and 1106 EAC cases as well as 1602 controls, all of German descent. In all cases the diagnosis of BE or EAC was histopathologically confirmed. Controls were a population‐based sample from the Heinz Nixdorf Recall (HNR) study, a population‐based cohort to study risk factors for cardiovascular diseases 9. All participants signed informed consent and the study was approved by ethics committees from the Universities of Bonn and Leipzig (Germany). Although none of the controls were diagnosed with EAC, they were not screened for Barrett's esophagus status. The use of unscreened controls may have led to a decrease in statistical power. However, as the prevalence of BE is only 1–2% in the general population 1, the power of the present association study should not have been substantially reduced by the use of unscreened controls 10. In BE, 171 cases were females and 371 were males, whereas 134 EAC cases were females and 972 EAC cases males. In controls, 802 were females and 800 were males.

In patients, genotyping of all three reported BE risk variants (rs3072, rs2701108, rs3784262) was done using the Sequenom MassARRAY iPlex Gold^®^ system (Sequenom, San Diego, USA). For quality control intra‐ and interplate duplicates were genotyped. In addition, negative controls (H_2_O) were added on each 384 well plate in order to exclude contamination. Clusterplot of each SNP was visually checked and manually corrected if necessary. In controls, genotypes for all three markers were obtained from Illumina's HumanOmniExpress BeadArrays (Illumina, San Diego, USA). The genome‐wide data of the control sample have been previously used in several GWAS on different traits 11, 12, 13.

All genotype data underwent different quality control steps, including Hardy‐Weinberg equilibrium *P *>* *0.001 and call rate >99%. Single‐marker association analyses including sex as covariate were performed separately for BE, EAC, and BE/EAC. In addition, for each of the three sample sets the presence of sex‐specific association was tested. SAS software (SAS 8.02; SAS Institute Inc, Cary, NC) was used for the quality control as well as the single‐marker association analyses.

## Results

Table 1 shows the results of the case–control comparison in BE, EAC, and in the combined sample. In BE we could replicate the association at rs3784262 near *ALDH1A2* with *P *=* *9.70 × 10^−04^ (RR = 0.79, Table 1), the same allele was disease‐conferring as previously reported 8. In addition, the same alleles at rs3072 near *GDF7* and at rs2701108 near *TBX5* that conferred BE risk in the previously published study 8 were more prevalent in patients than in controls (RR = 1.05 and RR = 0.87, respectively), although this was not significant (Table 1). In EAC we found association at rs3072 near *GDF7* with *P *=* *1.48 × 10^−03^ (RR = 1.20) and at rs2701108 near *TBX5* with *P *=* *2.47 × 10^−02^ (RR = 0.88, Table 1). Although the same allele at rs3784262 near *ALDH1A2* that confers BE risk was slightly more prevalent in patients than in controls, this association was not significant (*P *=* *1.30 × 10^−01^, RR = 0.92, Table 1). Given the association findings obtained in the separate analyses, we found significant associations at all three loci in the combined BE/EAC sample. SNP rs3072 at *GDF7* was disease associated with *P *=* *7.53 × 10^−03^ (RR = 1.15), rs2701108 at *TBX5* showed disease association with *P *=* *1.12 × 10^−02^ (RR = 0.88), and rs3784262 at *ALDH1A2* with *P *=* *8.06 × 10^−03^ (RR = 0.88, Table 1). At all three loci, we observed no evidence for sex‐specific BE or EAC risk effects (data not shown).

**Table 1:** Association results for the three previously identified BE risk SNPs 8 in 542 BE and 1106 EAC cases as well as 1602 controls of German descent

Phenotype	SNP	Chromosome	Position (bp)^a^	Allele^b^	MAF^c^ (%) in cases	MAF^c^ (%) in controls	RR^d^ (95% CI)	*P* value	Nearby gene^e^
BE	rs3072	2p24	20,741,887	G/A	38.1	37.0	1.05 (0.91–1.21)	5.32 × 10^−01^	*GDF7*
BE	rs2701108	12q24	113,158,644	G/A	35.5	38.6	0.87 (0.75–1.01)	6.38 × 10^−02^	*TBX5*
BE	rs3784262	15q22	56,040,398	G/A	40.7	46.4	0.79 (0.68–0.91)	9.70 × 10^−04^	*ALDH1A2*
EAC	rs3072	2p24	20,741,887	G/A	41.3	37.0	1.20 (1.07–1.34)	1.48 × 10^−03^	*GDF7*
EAC	rs2701108	12q24	113,158,644	G/A	35.6	38.6	0.88 (0.78–0.98)	2.47 × 10^−02^	*TBX5*
EAC	rs3784262	15q22	56,040,398	G/A	44.3	46.4	0.92 (0.82–1.03)	1.30 × 10^−01^	*ALDH1A2*
BE/EAC	rs3072	2p24	20,741,887	G/A	40.3	37.0	1.15 (1.04–1.27)	7.53 × 10^−03^	*GDF7*
BE/EAC	rs2701108	12q24	113,158,644	G/A	35.6	38.6	0.88 (0.79–0.97)	1.12 × 10^−02^	*TBX5*
BE/EAC	rs3784262	15q22	56,040,398	G/A	43.1	46.4	0.88 (0.79–0.97)	8.06 × 10^−03^	*ALDH1A2*

a Chromosomal position according to hg18.

b First allele represents the minor allele.

c Minor allele frequency (MAF) is given for cases and controls.

d Relative Risk (RR) with 95% Confidence Interval (CI) indicating the genetic effect size is given for the minor allele.

e Nearest gene to the associated SNPs is shown.

## Discussion

BE and EAC represent two stages within the esophagitis‐metaplasia‐dysplasia‐adenocarcinoma sequence. It has been shown that genetic risk factors are relevant in the etiology of BE and EAC. However, this does not necessarily mean that genetic variants confer equal risk to all different stages of the disease sequence. In this study, we analyzed three previously published BE variants 8 in BE and EAC samples from Germany in order to determine their risk effects during BE/EAC sequence. Our data show that rs3072 at *GDF7* and rs2701108 at *TBX5* are also conferring risk to EAC. Although the genetic risk effect of rs2701108 was similar in BE and EAC (RR = 0.87 and RR = 0.88, respectively), the risk effect of rs3072 was even higher in EAC compared to BE (RR = 1.20 and RR = 1.05, respectively). We therefore conclude that both loci are conferring disease risk also at later stages of the BE/EAC sequence. In contrast, rs3784262 at *ALDH1A2* was highly significantly BE associated (*P *=* *9.70 × 10^−04^), but showed no association with EAC (*P *=* *1.30 × 10^−01^), although the size of the latter sample was substantially larger (542 BE vs. 1106 EAC cases). Although many reasons may have led to an overestimated risk effect of rs3784262 in BE and an underestimated risk effect of this variant in EAC, our data do not provide evidence that this locus confer equal risk in early and late stages of the BE/EAC sequence.

Based on their genomic location and biological function**,** *GDF7* near rs3072, *TBX5* at rs2701108 and *ALDH1A2* near rs3784262 are all promising risk‐conferring genes for BE and EAC (summarized in Palles et al. 8). *GDF7* encodes the BMP12 protein and thereby function**s** in the PMP pathway that has been already implicated in BE development 14. Among various functions *TBX5* plays a role in the development of diaphragmatic musculature 15 and genetic variation influencing *TBX5* may predispose to hiatus hernia and thereby GERD. *ALDH1A2* encodes for an enzyme that catalyzes the synthesis of retinoic acid and may also be involved in alcohol metabolism 16 and hence be relevant for inflammation. Of note, alcohol consumption has been discussed as a risk factor for BE/EAC 17, 18. However, future studies (including animal models) have to show whether *GDF7*,* TBX5* and *ALDH1A2* represent the true risk‐conferring genes at the disease associated loci. Furthermore, GWAS have led to the identification of more than 7 BE and EAC risk variants within the past 3 years 4, 5, 6, 8. Aside from functional analyses in order to elucidate the pathophysiological mechanism at each implicated locus and to identify pathways in which risk genes are enriched, further GWAS and GWAS meta‐analyses on larger and detailed phenotyped sample sizes with BE and EAC are needed. This also will allow for mapping of all risk variants and genes in the BE/EAC sequence in order to identify biomarkers that predict EAC conversion in future.
